# Euthanasia and Physician-Assisted Suicide in People With an Accumulation of Health Problems Related to Old Age: A Cross-Sectional Questionnaire Study Among Physicians in the Netherlands

**DOI:** 10.3389/ijph.2024.1606962

**Published:** 2024-04-18

**Authors:** Frédérique W. M. Kraak-Steenken, Sophie C. Renckens, H. Roeline W. Pasman, Fenne Bosma, Agnes van der Heide, Bregje D. Onwuteaka-Philipsen

**Affiliations:** ^1^ Department of Public and Occupational Health, Amsterdam Public Health Research Institute, VU Medical Center, Amsterdam, Netherlands; ^2^ Expertise Center for Palliative Care Amsterdam UMC, Amsterdam, Netherlands; ^3^ Department of Public Health, Erasmus MC, University Medical Center, Rotterdam, Netherlands

**Keywords:** accumulation of health problems related to old age, euthanasia, physician-assisted suicide, end-of-life care, medical decision-making

## Abstract

**Objectives:** We explored characteristics of people with an accumulation of health problems related to old age requesting euthanasia or physician-assisted suicide (EAS) and identified characteristics associated with granting EAS requests.

**Methods:** We conducted a cross-sectional questionnaire study among Dutch physicians on characteristics of these people requesting EAS (*n* = 123). Associations between characteristics and granting a request were assessed using logistic regression analyses.

**Results:** People requesting EAS were predominantly >80 years old (82.4%), female (70.0%), widow/widower (71.7%), (partially) care-dependent (76.7%), and had a life expectancy >12 months (68.6%). The most prevalent health problems were osteoarthritis (70.4%) and impaired vision and hearing (53.0% and 40.9%). The most cited reasons to request EAS were physical deterioration (68.6%) and dependence (61.2%). 44.7% of requests were granted. Granting a request was positively associated with care dependence, disability/immobility, impaired vision, osteoporosis, loss of control, suffering without prospect of improvement and a treatment relationship with the physician >12 months.

**Conclusion:** Enhanced understanding of people with an accumulation of health problems related to old age requesting EAS can contribute to the ongoing debate on the permissibility of EAS in people without life-threatening conditions.

## Introduction

In the Netherlands, it is possible for citizens to request euthanasia or physician-assisted suicide (EAS). Since 2002, Dutch law states that it is not a criminal offense for a physician to perform euthanasia (physician administers lethal dose) or physician-assisted suicide (physician supplies the drug but the patient administers it him- or herself) if the due care criteria are met (Box 1) [1]. An example of one of those criteria is that, according to the physician, the patients’ suffering must be unbearable and without prospect of improvement. EAS is most often requested by patients with a life-threatening somatic condition, such as cancer [2]. However, EAS may also be requested by people suffering from non-life-threatening conditions.

BOX 1 **Due care criteria (Fourth evaluation of the Dutch euthanasia act, Netherlands, 2022) [1].**

1. The physician must be satisfied that the patient’s request is voluntary and well-considered.
2. The physician must be satisfied that the patient’s suffering is unbearable and without prospect of improvement.
3. The physician must have informed the patient about the patient’s situation and prognosis.
4. The physician must have come to the conclusion, together with the patient, that there is no reasonable alternative in the patient’s situation.
5. The physician must consult at least one other, independent physician, who must see the patient and give a written opinion on whether the statutory due care criteria have been fulfilled.
6. The physician must have exercised due medical care and attention in performing EAS.

There has been public debate about whether EAS requests from people without a life-threatening somatic condition can fall within the scope of the Dutch euthanasia law. In the renowned case of Mr. Brongersma, euthanasia was performed because he was “tired of living.” On appeal, the court concluded that Mr. Brongersma’s suffering had no medical grounds and therefore did not fall within the scope of the law [3]. However, of those who consider their life “completed” (are “tired of living”) and request EAS, many actually have medical complaints such as an accumulation of health problems related to old age [4–9]. Health problems related to old age include, amongst others, vision and hearing impairments, osteoporosis, osteoarthritis, balance problems and cognitive decline. In recent years, it has become clear that for some people, health problems related to old age and the subsequent limitations can result in unbearable suffering without prospect [2, 10–14]. Therefore, a request from an older adult with an accumulation of health problems related to old age can be granted under the current law, as long as there is a predominant medical ground for the suffering and all criteria of due care are met [5, 9, 15].

It is likely that the number of requests from people suffering from an accumulation of health problems related to old age will increase as life expectancy increases. This is also shown from the data of the Dutch Regional Review Committees (RTE) of recent years. In 2015, the total number of granted requests of people with an accumulation of health problems related to old age was 183, and in 2022, it increased to 379 granted requests [16, 17]. Nonetheless, this is only a small fraction (4.3%) of the total number of granted requests that were reported to the RTE [17]. This can possibly be related to the relatively low number of EAS requests in the case of an accumulation of health problems related to old age, but also to the reluctance of physicians to grant such requests. The fourth evaluation of the Dutch euthanasia act showed that 55% of the physicians found it conceivable to perform EAS based on an accumulation of health problems related to old age (of which 14% had performed EAS in such a case) [18]. This is lower than for EAS in general (resp. 82% and 55%) [18]. Another study in deceased patients showed that of the people with an accumulation of health problems related to old age, 8% of them requested EAS, which was granted in 46% of the cases. The most common reason for refusal was that there was no unbearable suffering according to the physician [12]. Schnabel et al. [5] also stated that physicians are not always well informed about the possibilities offered by the current law, such as that this law provides a possibility for termination of life in the event of suffering due to an accumulation of health problems related to old age.

Some people could be refused EAS (unfairly) because the physician is not well informed about the law’s possibilities.

To our knowledge, there is still little information available about the characteristics of these people with an accumulation of health problems related to old age who request EAS. Given the continued increase in EAS requests from people with an accumulation of health problems related to old age, it is important to gain more insight into the characteristics of these people.

Therefore, we aim to explore the following research questions: What are the characteristics of people with an accumulation of health problems related to old age who requested EAS? What does the decision-making process of physicians on whether to grant or refuse the request look like? Which characteristics of physicians, people requesting EAS, and the decision-making process are associated with granting an EAS request from people with an accumulation of health problems related to old age?

## Methods

### Design and Population

This cross-sectional study consisted of an online questionnaire sent to a total of 2,500 physicians in the Netherlands [18]. For a random sample, postal addresses of 1,100 general practitioners, 400 elderly care physicians, and 1,000 clinical specialists (consisting of cardiologists, pulmonologists, internists, neurologists, surgeons and intensive care physicians) were obtained via the national database of registered physicians (IQVIA) [19].

The inclusion criteria for physicians were as follows: 1) having been working in patient care in the Netherlands for the past year and 2) having a registered work or home address in IQVIA.

### Data Collection

Data were collected from April until September 2022.

A letter with information about the study and a link to the online questionnaire was sent to the physicians’ postal address, followed by two reminders (with an interval of 3 weeks). The second reminder also included an abbreviated two-page paper version of the questionnaire containing only the most essential questions.

The questionnaire consisted of questions about EAS with the following subjects: a) characteristics of the physician (demographic and professional characteristics), b) experiences with EAS requests and their performance, c) characteristics of the last received request for EAS in the past 5 years and d) opinion on a number of statements.

In this study, the focus is on part c of the questionnaire. There were multiple different versions of part c, namely, about an EAS request of a person with 1) dementia; 2) an accumulation of health problems related to old age; and 3) another condition. Based on their experience with these particular conditions, physicians were directed to one of the versions; they only completed one version (See Supplementary File S1).

The questions about last received request for EAS of a person with an accumulation of health problems related to old age included the following: existing health problems related to old age, other characteristics of people with an accumulation of health problems related to old age who requested EAS (e.g., gender, age, living situation, level of dependency, life expectancy and main reason for requesting EAS), duration of treatment relationship, period between first conversation about EAS and explicit request, duration of the decision-making process after a request and whether the physician had granted or refused the request. In the event that a request was refused, the physician was asked about the reasons why the request was refused and what the subsequent treatment was. Demographics and professional characteristics of the physician were also included (e.g., gender, age, religion, medical specialty, years of experience, additional training, being a palliative care consultant or SCEN physician, familiarity with the Dutch Euthanasia Code (See Supplementary File S2 for more detailed explanations of the variables).

### Analyses

Statistical analyses were carried out using IBM SPSS 28. Descriptive statistics were used to describe the characteristics of respondents and the characteristics of people with an accumulation of health problems related to old age who requested EAS and the decision-making process. Regression analyses were performed to determine the association between the dependent variable *request granted* (versus refused as reference group) and the characteristics of physicians, people requesting EAS, and the decision-making process as independent variables. First, univariable regression analysis was used to determine the association of the variables individually. Then, the variables with a *p*-value below 0.10 were included in a multivariable logistic regression analysis using manual stepwise backward selection (removal at *p* > 0.05). Which person characteristics were associated with granting an EAS request in case of an accumulation of health problems related to old age was identified by three separate multivariable logistic regression analyses considering the small sample size (*n* = 123 case reports). The odds ratios (ORs) were calculated with a 95% confidence interval. Before adding the variables in the multivariable regression, we checked that they were not highly correlated with each other using the Pearson correlation coefficient. One variable was excluded from the multivariable analysis due to a high correlation (Table 3). The other Pearson correlation coefficients were below 0.5.

## Results

### Characteristics of Respondents

Of a total of 2,500 physicians who were invited to participate, 2,255 met the inclusion criteria. The response rate was 33% (*n* = 746). Of these physicians, 123 answered questions about a person with an accumulation of health problems related to old age who requested them to perform EAS (a case from the past 5 years).

Table 1 provides an overview of the background and professional characteristics of the physicians who answered questions about a person with an accumulation of health problems related to old age who requested EAS. The respondents consisted of 93 general practitioners (GPs), 25 elderly care physicians (ECPs) and 4 clinical specialists (2 internists, 1 intensivist and 1 cardiologist). Of the respondents, 56.6% were male, 56.6% were 51 years or older, 35.2% were religious, 5.0% were palliative care consultants, 5.0% were SCEN physicians and 6.6% had received certified palliative care training.

**TABLE 1:** Background characteristics of respondents (Fourth evaluation of the Dutch euthanasia act, Netherlands, 2022).

	GPs (*n* = 93) (%)	ECPs (*n* = 25) (%)	Clinical specialists (*n* = 4) (%)	Total (*n* = 123)^a^ (%)
Age				
<41 years	19.4	4.0	0.0	15.6
41–50 years	30.1	24.0	0.0	27.9
51–60 years	36.6	40.0	50.0	37.7
>60 years	14.0	32.0	50.0	18.9
Gender				
Male	53.8	60.0	100.0	56.6
Female	46.2	40.0	0.0	43.4
Religious	26.9	60.0	75.0	35.2
Years of work experience (in their medical specialty)				
<11 years	17.2	0.0	0.0	13.1
11–20 years	33.3	20.0	0.0	29.5
21–30 years	35.5	48.0	50.0	38.5
>30 years	14.0	32.0	50.0	18.9
Additional functions				
Palliative care consultant	4.3	8.3	0.0	5.0
SCEN physician^b^	5.4	4.2	0.0	5.0
Certified palliative care training^c^	5.4	12.0	0.0	6.6
Number of deceased patients in the last year				
<10 patients	15.4	12.0	0.0	14.3
10–14 patients	26.4	20.0	33.3	25.2
15–25 patients	34.1	36.0	0.0	33.6
>25 patients	24.2	32.0	66.7	26.9
Ever consulted the Dutch Euthanasia Code^d^	87.0	80.0	100.0	86.0

EAS, Euthanasia and/or physician-assisted suicide; GPs, general practitioners; ECPs, elderly care physicians.

Missing values: Physician group 0.8%; Palliative care consultant 0.8%; SCEN physician 0.8%; Number of deceased patients in the last year 2.4%; Ever consulted the Dutch Euthanasia Code 2.2%.

^a^ One respondent did not specify their medical specialty, hence the number of the total group is one respondent higher.

^b^ A SCEN physician is a trained physician from whom other physicians can obtain information and advice about EAS, or request a formal consultation (one of the criteria of due care).

^c^ Not including regular curricular training.

^d^ This question was only asked in the online questionnaire, to physicians who indicated that they were familiar with the Dutch Euthanasia Code (*n* = 89). The Dutch Euthanasia Code specifies the due care criteria against which the Regional Euthanasia Review Committee assesses EAS notifications.

### Characteristics of People With an Accumulation of Health Problems Related to Old Age Who Requested EAS and of the Decision-Making Process

Of the 123 people with an accumulation of health problems related to old age who had requested EAS, 55 (44.7%) had their request granted, and 68 (55.3%) had their request denied (Table 2). In both of these groups, the majority were between 80 and 89 years of age (resp. 61.1%; 47.7%) and of female gender (resp. 72.2%; 68.2%). Furthermore, in both groups, osteoarthritis (resp. 66.7%; 73.8%), vision impairment (resp. 66.7%; 41.0%) and hearing impairment (resp. 46.3%; 36.1%) were the most common health problems related to old age.

**TABLE 2:** Characteristics of cases in which a person with an accumulation of health problems related to old age requested euthanasia or physician-assisted suicide (Fourth evaluation of the Dutch euthanasia act, Netherlands, 2022).

	EAS performed (*n* = 55) (%)	EAS request declined (*n* = 68) (%)	Total (*n* = 123) (%)
Person characteristics			
Age			
<80 years	11.1	23.1	17.6
80–89 years	61.1	47.7	53.8
≥90 years	27.8	29.2	28.6
Gender			
Male	27.8	31.8	30.0
Female	72.2	68.2	70.0
Health problems related to old age^a^			
Osteoarthritis	66.7	73.8	70.4
Vision impairment	66.7	41.0	53.0
Hearing impairment	46.3	36.1	40.9
Balance problems	40.7	32.8	36.5
Cognitive decline	24.1	27.9	26.1
Osteoporosis	29.6	9.8	19.1
Other	63.0	24.6	42.6
Overall decline	29.6	9.8	19.1
Heart failure/cardiac symptoms	24.1	4.9	13.9
Other	9.3	9.8	9.6
Main reason/reasons for EAS request^a^			
Physical decline	81.8	57.6	68.6
Dependency	70.9	53.0	61.2
(Fear of) losing control of one’s own life	63.6	42.4	52.1
General weakness/fatigue	65.5	39.4	51.2
Loss of dignity	54.5	33.3	43.0
Tired of living	41.8	42.4	42.1
Suffering with no prospect of improvement	65.5	21.2	41.3
Disability/immobility	49.1	31.8	39.7
No purpose in life	25.5	51.5	39.7
Pain	50.9	25.8	37.2
Loneliness	20.0	33.3	27.3
Other physical complaints	29.1	24.2	26.4
Not wanting to be a burden to family/environment	14.5	28.8	22.3
No longer living independent	21.8	21.2	21.5
Depressed feelings	12.7	27.3	20.7
Cognitive decline	12.7	13.6	13.2
Dyspnea	18.2	3.0	9.9
Fear	1.8	10.6	6.6
Death of a loved one	5.5	5.0	5.2
Other^b^	1.8	1.5	1.7
Partner			
No	1.9	16.7	10.0
Yes	13.0	22.7	18.3
Widow/widower	85.2	60.6	71.7
Place of residence last 3 months			
At home or with loved ones	63.6	62.1	62.8
Institutionalized	36.4	37.9	37.2
Dependency			
Independent	14.5	30.8	23.3
Limited care dependent	27.3	36.9	32.5
Care-dependent	58.2	32.3	44.2
Life expectancy			
Up to 12 months	45.5	19.7	31.4
More than 12 months	54.5	80.3	68.6
Treatment relationship			
Duration of treatment relationship			
Up to 12 months	14.5	30.8	23.3
More than 12 months	85.5	69.2	76.7
Decision-making process			
Period between first EAS conversation and explicit request			
<1 month	11.5	15.4	13.5
1 month–6 months	46.2	40.4	43.3
7 months–1 year	9.6	21.2	15.4
>1 year	32.7	23.1	27.9
Duration of the decision-making process			
<1 month	43.6	54.7	49.6
1–3 months	45.5	26.6	35.3
>3 months	10.9	18.8	15.1
Chosen performance			
Performed euthanasia or physician-assisted suicide^c^			
Euthanasia	96.4	—	96.4
Physician-assisted suicide	3.6	—	3.6
Reasons for refusing EAS and the subsequent treatment			
Reasons for declined EAS^d^			
Physician never performs EAS	—	13.6	13.6
Physician thought not all criteria of due care might be met	—	42.4	42.4
No unbearable suffering without prospect	—	16.7	16.7
Treatment options still available	—	6.1	6.1
Non-empathetic for suffering	—	4.5	4.5
No life-threatening condition/terminal	—	3.0	3.0
Other	—	12.1	12.1
Personal objections	—	43.9	43.9
Non-empathetic for suffering	—	19.7	19.7
Treatment options still available	—	1.5	1.5
Other	—	22.7	22.7
Treatment after declined EAS^d^			
No change in treatment	—	51.5	51.5
Curative treatment stopped	—	9.1	9.1
Symptom management	—	22.7	22.7
Discussed treatment limitation	—	30.3	30.3
Psychological counselling	—	21.2	21.2
Expansion of social activities	—	9.1	9.1
Expansion of care	—	19.7	19.7
Palliative sedation	—	3.0	3.0
Transfer to hospice	—	1.5	1.5
Transfer to other care facility	—	10.6	10.6
Discharge home	—	3.0	3.0
Home care started/expanded	—	10.6	10.6
Other	—	6.1	6.1
Consciously stop eating and drinking	—	4.5	4.5
Other	—	1.5	1.5

EAS, Euthanasia and/or physician-assisted suicide.

Missing values: Age 3.3%; Gender 2.4%; Health problems related to old age 6.5%; Main reason/reasons for EAS request 1.6%; Partner 2.4%; Place of residence 1.6%; Dependency 2.4%; Life expectancy 1.6%; Duration of treatment relationship 2.4%; Period between first EAS conversation and explicit request 15.4%; Duration of the decision-making process 3.3%; Reasons for declined EAS 1.5%; Treatment after declined EAS 2.9%.

^a^ Multiple answers possible.

^b^ The following reasons were cited: “Due to a disability the person could not do the activities she enjoyed and derived satisfaction from” and “grief over a family quarrel.”

^c^ This question was only asked to physicians who indicated that they had performed EAS after a person with an accumulation of health problems related to old age had requested EAS (*n* = 55).

^d^ This question was only asked to physicians who indicated that they had declined an EAS request from a person with an accumulation of health problems related to old age (*n* = 68).

Among the people whose EAS request was granted, physical decline (81.8%), dependency (70.9%), general weakness, and suffering with no prospect of improvement (both 65.5%) were the most frequently cited reasons for the request. Physical decline (57.6%), dependency (53.0%) and no purpose in life (51.5%) were most frequently cited as reasons for the EAS request among the group with people where the EAS request was refused.

Most people whose EAS request was granted had a treatment relationship with their physician for more than 12 months (85.5%) (Table 2). This also applied to a lesser extent in the group where the request had been refused (69.2%). Most cases where the EAS was granted involved euthanasia (96.4%), and 3.6% involved physician-assisted suicide.

Of the physicians who refused the EAS request, 13.6% did so because they never performed EAS, 42.4% because they thought that not all due care criteria were met (e.g., no unbearable suffering without prospect, treatment options still available and non-empathetic for suffering), and 43.9% because of personal objections (e.g., non-empathetic for suffering). Some physicians (3.0%) expressed the belief that the due care criteria could not be met because of the absence of a life-threatening disease or terminal condition. In 51.5% of the cases, there was no change in treatment after refusal. In other cases, there were discussions about treatment limitations (30.3%), initiation of symptom management (22.7%), or psychological counselling (21.2%).

### Physician Characteristics Associated With Granting an EAS Request in Case of an Accumulation of Health Problems Related to Old Age

Physicians between the ages of 51–60 years and 61 years and older were more likely to perform EAS in case of an accumulation of age-related health problems compared with physicians younger than 41 years (51–60 years old: OR 4.48 [1.15–17.50]; 61 years and older: OR 16.00 [3.43–74.70]) (Table 3). No other physician characteristics were found to be significantly associated in the multivariable regression analysis.

**TABLE 3:** Association between physician characteristics and a granted euthanasia or physician-assisted suicide request in case of an accumulation of health problems related to old age (*n* = 123; Fourth evaluation of the Dutch euthanasia act, Netherlands, 2022).

	Performed EAS in case of an accumulation of health problems related to old age		
	Row percentage (%)	Univariable	Multivariable^a^
		OR (95% CI)	OR (95% CI)^a^
Age			
<41 years (*n* = 19)	15.8	Reference	Reference
41–50 years (*n* = 34)	38.2	**3.30 (0.80–13.58)**	3.30 (0.80–13.58)
51–60 years (*n* = 46)	45.7	**4.48 (1.15–17.50)**	**4.48 (1.15–17.50)**
>60 years (*n* = 24)	75.0	**16.00 (3.43–74.70)**	**16.00 (3.43–74.70)**
Gender			
Male (*n* = 70)	52.9	Reference	a
Female (*n* = 53)	34.0	**0.46 (0.22–0.96)**	a
Medical specialty			
GPs (*n* = 93)	46.2	Reference	—
ECPs (*n* = 25)	32.0	0.55 (0.22–1.39)	—
Clinical specialists (*n* = 4)	75.0	3.49 (0.35–34.78)	—
Religious			
No (*n* = 80)	46.3	Reference	—
Yes (*n* = 43)	41.9	0.84 (0.40–1.77)	—
Years of work experience (in their medical specialty)			
<11 years (*n* = 16)	12.5	Reference	b
11–20 years (*n* = 36)	47.2	**6.26 (1.24–31.64)**	b
21–30 years (*n* = 47)	44.7	**5.65 (1.15–27.71)**	b
>30 years (*n* = 24)	62.5	**11.67 (2.14–63.64)**	b
Palliative care consultant			
No (*n* = 116)	44.0	Reference	—
Yes (*n* = 6)	66.7	2.55 (0.45–14.47)	—
Number of deceased patients in the last year			
<10 patients (*n* = 17)	52.9	Reference	—
10–14 patients (*n* = 30)	33.3	0.44 (0.13–1.50)	—
15–25 patients (*n* = 41)	46.3	0.77 (0.25–2.38)	—
>25 patients (*n* = 32)	50.0	0.89 (0.27–2.89)	—
Ever consulted the Dutch Euthanasia Code			
No (*n* = 12)	50.0	Reference	—
Yes (*n* = 75)	46.7	0.88 (0.26–2.96)	—

Euthanasia and/or physician-assisted suicide (EAS), general practitioners (GPs), elderly care physicians (ECPs), odds ratio (OR), confidence interval (CI).

Missing values: Physician group 0.8%; Palliative care consultant 0.8%; Number of deceased patients in the last year 2.4%; Ever consulted the Dutch Euthanasia Code 2.2%.

The variable SCEN physician was not included in the regression due to a very large range of the confidence interval.

Odds ratios in bold in univariable regression means the a *p*-value is < 0.10, in multivariable regression it means the *p*-value is < 0.05.

^a^ Some variables were entered in the first step of the backward regression, however, later removed due to a *p*-value below 0.05.

^b^ Because age and work experience were strongly correlated (Pearson = 0.80), work experience was excluded from the multivariable analysis.

### Person Characteristics Associated With Granting an EAS Request in Case of an Accumulation of Health Problems Related to Old Age

Persons with an accumulation of health problems related to old age had a lower probability that their EAS request was granted when they had no partner compared to people who were widow/widower (OR 0.09 [0.01–0.82]) (Table 4). In addition, being care-dependent increased the likelihood that the physician would grant EAS for such a request compared with people who were independent (OR 10.07 [3.00–33.78]). Furthermore, a treatment relationship duration of more than 12 months increased the likelihood that the physician would grant EAS for such a request (OR 5.65 [1.75–18.21]).

**TABLE 4:** Association between person characteristics and a granted euthanasia or physician-assisted suicide request in case of an accumulation of health problems related to old age (*n* = 123; Fourth evaluation of the Dutch euthanasia act, Netherlands, 2022).

	Performed EAS in case of an accumulation of health problems related to old age		
	Row percentage (%)	Univariable	Multivariable
		OR (95% CI)	OR (95% CI)^a^
Age			
<80 years (*n* = 21)	28.6	Reference	a
80–89 years (*n* = 64)	51.6	**2.66 (0.92–7.73)**	a
≥90 years (*n* = 34)	44.1	1.97 (0.62–6.32)	a
Gender			
Male (*n* = 36)	41.7	Reference	—
Female (*n* = 84)	46.4	1.21 (0.55–2.67)	—
Partner			
Widow/widower (*n* = 86)	53.5	Reference	Reference
Yes (*n* = 22)	31.8	**0.41 (0.15–1.09)**	0.36 (0.12–1.09)
No (*n* = 12)	8.3	**0.08 (0.01–0.64)**	**0.09 (0.01–0.82)**
Place of residence last 3 months			
At home or with loved ones (*n* = 76)	46.1	Reference	—
Institutionalized (*n* = 45)	44.4	0.94 (0.45–1.97)	—
Dependency			
Independent (*n* = 28)	28.6	Reference	Reference
Limited care dependent (*n* = 39)	38.5	1.56 (0.55–4.44)	1.80 (0.60–5.40)
Care-dependent (*n* = 53)	60.4	**3.81 (1.42–10.23)**	**10.07 (3.00–33.78)**
Life expectancy			
Up to 12 months (*n* = 38)	65.8	Reference	a
More than 12 months (*n* = 83)	36.1	**0.29 (0.13–0.66)**	a
Duration of treatment relationship			
Up to 12 months (*n* = 28)	28.6	Reference	Reference
More than 12 months (*n* = 92)	51.1	**2.61 (1.05–6.53)**	**5.65 (1.75–18.21)**

Euthanasia and/or physician-assisted suicide (EAS), general practitioners (GPs), elderly care physicians (ECPs), odds ratio (OR), confidence interval (CI).

Missing values: Age patient 3.3%; Gender patient 2.4; Partner 2.4%; Place of residence last 3 months 1.6%; Dependency 2.4%; Life expectancy 1.6%; Duration of treatment relationship 2.4%.

Odds ratios in bold in univariable regression means the a *p*-value is < 0.10, in multivariable regression it means the *p*-value is < 0.05.

^a^ Some variables were entered in the first step of the backward regression, however, later removed due to a *p*-value below 0.05.

Table 5 shows the associations between the type of health problems related to old age and a granted EAS request. Having a vision impairment (OR 5.65 [2.03–15.69]) or osteoporosis (OR 5.81 [1.74–19.40]) gave an increased likelihood of a granted EAS request.

**TABLE 5:** Association between peoples’ type of health problems related to old age and a granted euthanasia or physician-assisted suicide request in case of an accumulation of health problems related to old age (*n* = 123; Fourth evaluation of the Dutch euthanasia act, Netherlands, 2022).

	Performed EAS in case of an accumulation of health problems related to old age		
	Row percentage (%)	Univariable	Multivariable
		OR (95% CI)	OR (95% CI)^a^
Health problems related to old age			
Osteoarthritis			
No (*n* = 34)	52.9	Reference	—
Yes (*n* = 81)	44.4	0.71 (0.32–1.59)	—
Vision impairment			
No (*n* = 54)	33.3	Reference	Reference
Yes (*n* = 61)	59.0	**2.88 (1.34–6.17)**	**5.65 (2.03–15.69)**
Hearing impairment			
No (*n* = 68)	42.6	Reference	—
Yes (*n* = 47)	53.2	1.53 (0.72–3.23)	—
Balance problems			
No (*n* = 73)	43.8	Reference	—
Yes (*n* = 42)	52.4	1.41 (0.66–3.02)	—
Cognitive decline			
No (*n* = 85)	48.2	Reference	—
Yes (*n* = 30)	43.3	0.82 (0.36–1.90)	—
Osteoporosis			
No (*n* = 93)	40.9	Reference	Reference
Yes (*n* = 22)	72.7	**3.86 (1.38–10.76)**	**5.81 (1.74–19.40)**
Other			
No (*n* = 66)	30.3	Reference	Reference
Yes (*n* = 49)	69.4	**5.21 (2.34–11.64)**	**11.53 (4.02–33.10)**

EAS, Euthanasia and/or physician-assisted suicide; GPs, general practitioners; ECPs, elderly care physicians, odds ratio (OR), confidence interval (CI).

Missing values: Health problems related to old age 6.5%.

Odds ratios in bold in univariable regression means the a *p*-value is < 0.10, in multivariable regression it means the *p*-value is < 0.05.

^a^No variables were removed after the first step of the backward regression because the *p*-values were below 0.05.

Furthermore, if the person reported losing control over one’s own life (or feared this) (OR 3.84 [1.51–9.77]), suffered without prospect of improvement (OR 8.22 [3.16–21.36]), or had a disability/immobility (OR 2.77 [1.02–7.50]), there was a higher likelihood of a granted EAS request (Table 6). In contrast, no purpose in life lowered the likelihood of a granted EAS request (OR 0.13 [0.04–0.40]). Not wanting to be a burden to the family/environment also had a smaller likelihood that the physician would grant an EAS request in case of an accumulation of health problems related to old age (OR 0.30 [0.09–0.95]).

**TABLE 6:** Association between reason for the euthanasia or physician-assisted suicide request and a granted euthanasia or physician-assisted suicide request in case of an accumulation of health problems related to old age (*n* = 123; Fourth evaluation of the Dutch euthanasia act, Netherlands, 2022).

	Performed EAS in case of an accumulation of health problems related to old age		
	Row percentage (%)	Univariable	Multivariable
		OR (95% CI)	OR (95% CI)^a^
Main reason/reasons for EAS request			
Physical decline			
No (*n* = 38)	26.3	Reference	a
Yes (*n* = 83)	54.2	**3.32 (1.43–7.69)**	a
Dependency			
No (*n* = 47)	34.0	Reference	a
Yes (*n* = 74)	52.7	**2.16 (1.01–4.60)**	a
(Fear of) losing control of one’s own life			
No (*n* = 58)	34.5	Reference	Reference
Yes (*n* = 63)	55.6	**2.38 (1.14–4.95)**	**3.84 (1.51–9.77)**
General weakness/fatigue			
No (n = 59)	32.2	Reference	a
Yes (n = 62)	58.1	**2.92 (1.39–6.13)**	a
Loss of dignity			
No (*n* = 69)	36.2	Reference	a
Yes (*n* = 52)	57.7	**2.40 (1.15–5.02)**	a
Tired of living			
No (*n* = 70)	45.7	Reference	—
Yes (*n* = 51)	45.1	0.98 (0.47–2.01)	—
Suffering with no prospect of improvement			
No (*n* = 71)	26.8	Reference	Reference
Yes (*n* = 50)	72.0	**7.04 (3.13–15.83)**	**8.22 (3.16–21.36)**
Disability/immobility			
No (*n* = 73)	38.4	Reference	Reference
Yes (*n* = 48)	56.3	**2.07 (0.99–4.33)**	**2.77 (1.02–7.50)**
No purpose in life			
No (*n* = 73)	56.2	Reference	Reference
Yes (*n* = 48)	29.2	**0.32 (0.15–0.70)**	**0.26 (0.10–0.70)**
Pain			
No (*n* = 76)	35.5	Reference	a
Yes (*n* = 45)	62.2	**2.99 (1.39–6.42)**	a
Loneliness			
No (*n* = 88)	50.0	Reference	—
Yes (*n* = 33)	33.3	0.50 (0.22–1.15)	—
Other physical complaints			
No (*n* = 89)	43.8	Reference	—
Yes (*n* = 32)	50.0	1.28 (0.57–2.88)	—
Not wanting to be a burden to family/environment			
No (*n* = 94)	50.0	Reference	Reference
Yes (*n* = 27)	29.6	**0.42 (0.17–1.06)**	**0.30 (0.09–0.95)**
No longer living independent			
No (*n* = 95)	45.3	Reference	—
Yes (*n* = 26)	46.2	1.04 (0.43–2.48)	—
Depressed feelings			
No (*n* = 96)	50.0	Reference	a
Yes (*n* = 25)	28.0	**0.39 (0.15–1.02)**	a
Cognitive decline			
No (*n* = 105)	45.7	Reference	—
Yes (*n* = 16)	43.8	0.92 (0.32–2.67)	—
Dyspnea			
No (*n* = 109)	41.3	Reference	a
Yes (*n* = 12)	83.3	**7.11 (1.49–34.02)**	a
Fear			
No (*n* = 113)	47.8	Reference	—
Yes (*n* = 8)	12.5	0.16 (0.02–1.31)	—
Death of a loved one			
No (*n* = 115)	45.2	Reference	—
Yes (*n* = 6)	50.0	1.21 (0.24–6.26)	—
Other			
No (*n* = 119)	45.4	Reference	—
Yes (*n* = 2)	50.0	1.20 (0.07–19.70)	—

EAS, Euthanasia and/or physician-assisted suicide; GPs, general practitioners; ECPs, elderly care physicians, odds ratio (OR), confidence interval (CI).

Missing values: Main reason/reasons for EAS, request 1.6%.

Odds ratios in bold in univariable regression means the a *p*-value is < 0.10, in multivariable regression it means the *p*-value is < 0.05.

^a^ Some variables were entered in the first step of the backward regression, however, later removed due to a *p*-value below 0.05.

## Discussion

Among the people who made an EAS request, care dependence and loss of control played an important role. These characteristics were also positively associated with physicians granting a request. In addition, longer treatment relationship, type of health problem related to old age, disability/immobility and suffering without prospect of improvement seem to be important in their decision to grant an EAS request.

### People With an Accumulation of Health Problems Related to Old Age Requesting EAS

People who requested EAS because of an accumulation of health problems related to old age most often had osteoarthritis, vision impairment and/or hearing impairment. They frequently named physical decline, dependency, (fear of) losing control of one’s own life and/or general weakness/fatigue as reasons for the request. The majority had a life expectancy of more than 12 months. In the study by Van Wijngaarden et al. [20], it emerges that older adults with a desire to die have, among other things, an aversion to becoming dependent. This might explain why especially people with vision and hearing impairment and/or osteoarthritis request EAS, since these health problems related to old age are often accompanied by (increasing) dependence [21–23].

In our study, many physicians who refused an EAS request in case of an accumulation of health problems related to old age indicated that they could not empathize with the person’s suffering or indicated that they did not consider the suffering as unbearable and without prospect. This may be because their personal boundaries differ from the possibilities offered by the law. It is permissible for a physician to refuse a request due to personal objections and do not use the possible space provided by the Dutch euthanasia law. However, it is not desirable for a physician to refuse an EAS request if this is based on incorrect beliefs. Our research showed that some physicians refused an EAS request based on the incorrect belief that performing EAS in a person without a life-threatening condition does not fall within the scope of the Dutch euthanasia. Other research also cited incorrect beliefs of physicians about the Dutch euthanasia law (e.g., the life expectancy should be less than 2 weeks) [24]. This could indicate a lack of knowledge about the Dutch euthanasia law. Schnabel et al. [5] also indicated that it was noted from their focus groups with (SCEN) physicians that not all physicians are aware that people with an accumulation of health problems related to old age are eligible for EAS. Increasing awareness among physicians about the scope of the law could possibly prevent physicians from refusing EAS due to incorrect beliefs.

### Characteristics Associated With Granting an EAS Request in Case of an Accumulation of Health Problems Related to Old Age

Few physician characteristics were associated with granting an EAS request. Of the person characteristics, the type of health problem related to old age (vision impairment and osteoporosis), certain reasons for EAS [(fear of) losing control of one’s own life, suffering with no prospect of improvement and disability/immobility], dependency and longer treatment relationship with the physician were positively associated with the likelihood of a granted EAS request. Regarding the treatment relationship, Ten Cate et al. [24] showed that personal preferences of physicians emerged when performing EAS, such as the desire to know the person well before performing EAS. This reasoning could explain the result of a positive association between a longer treatment relationship and the granting of an EAS request found in our study.

People with an accumulation of health problems related to old age who had no partner were less likely to have their EAS request granted. Furthermore, having “no purpose in life” and/or “not wanting to be a burden to family/environment” as a reason for an EAS request lowered the likelihood of a granted EAS request. This seems to be in line with the finding of Pasman et al. [25] that physicians put more emphasis on physical suffering in an EAS request. These reasons for requesting EAS could raise doubts for the physician as to whether the due care criteria can be met, as an EAS request without suffering based on a medical condition is not allowed. However, it is a thin line, where the wording of the request is important in assessing whether the person is suffering “from” an accumulation of health problems related to old age or “with” an accumulation of health problems related to old age. It is, for instance, possible that “no purpose in life” roots from being limited in daily living due to the health problems related to old age.

### International Perspective

Abroad, euthanasia has also been legalized in several jurisdictions, including Spain, Belgium, Luxembourg, Canada, Colombia, six states in Australia, and New Zealand [18, 26, 27]. Belgium runs almost parallel to the Netherlands in terms of legislation and the gradual expansion of its interpretation [26]. Cases of people with an accumulation of health problems related to old age who requested EAS have also occurred here. Canada recently (2021) amended legislation (Bill C-14 to Bill C-7), removing “reasonably foreseeable death” as an eligibility criterion, allowing EAS in people with an accumulation of health problems related to old age [11]. However, in Colombia, Australia and New Zealand, legislation is limited to those with a terminal illness [26]. The study by Mroz et al. [26] describes that in some countries where EAS has been legalized for some time (e.g., in the Netherlands and in Belgium), a process of conceptual gradual “filling” of the existing legal space was observed. This means that EAS initially was granted to people who were most obviously eligible (e.g., those who are terminally ill) and then gradually expanded to groups of people who were less obviously eligible (non-terminal people and people without a life-threatening illness). Thus, it is arguable that more knowledge on this topic (e.g., knowledge on EAS in people with an accumulation of health problems related to old age) in other countries can also influence policies, medical practices and societal attitudes.

### Strengths and Limitations

The most important strengths of this study are that it used a randomized sample of Dutch physicians practicing within different specialties. Their opinions and actions on EAS requests in case of an accumulation of health problems related to old age were investigated, a still relatively unknown topic within science. More awareness on this topic is important for the ongoing debate on EAS in this group of people. Furthermore, the questionnaire was anonymous, reducing the likelihood that physicians would give a socially desirable answer.

One possible limitation is recall bias, as questions were asked about past years. For example, when asking about a case from the past 5 years, possibly not everything could be read back in the patient files. Another limitation was that there was a small sample size for the questions about the cases (*n* = 123), which affected the analyses, especially in the multiple regression analysis, where not all variables could be included (simultaneously). Finally, the study focused only on the physician’s perspective, not that of a person with an accumulation of health problems related to old age. Research from this other perspective will provide more insight into what drove these people to request EAS (in addition to what the physician believes drove them). This information may enrich the ongoing debate.

### Conclusion

Multiple characteristics of people with an accumulation of health problems related to old age can affect the likelihood that an EAS request will be granted. Most of the reasons for EAS requests among people with an accumulation of health problems related to old age and factors positively associated with granting a request seem to be related to dependency and loss of control.

With the help of this study, there is a better understanding of which people request EAS in the case of an accumulation of health problems related to old age, which may be helpful in further public debate about what should and should not fall within the Dutch euthanasia law.

## Abbreviations

EAS, Euthanasia or physician-assisted suicide; RTE, The Dutch Regional Review Committees; SCEN physician, (Support and Consultation on Euthanasia in the Netherlands), a SCEN physician is a trained physician from whom other physicians can obtain information and advice about euthanasia or physician-assisted suicide, or request a formal consultation (one of the criteria of due care); GPs, General practitioners; ECPs, Elderly care physicians; OR, Odds ratio.

## References

[1] Dutch department of justice. The Dutch Termination of Life on Request and Assisted Suicide (Review Procedures) Act. Ethical Perspect (2002) 9(2):176–81. 15712446 10.2143/ep.9.2.503855

[2] Onwuteaka-Philipsen BD Legemaate J van der Heide A Van Delden JJM Evenblij K Hammoud I Derde evaluatie:Wet toetsing levensbeëindiging op verzoek en hulp bij zelfdoding [Third Evaluation: Law on the Review of the Termination of Life on Request and Assistance With Suicide]. Report (2017). 16223080

[3] Court of Appeal Amsterdam. ECLI:NL:GHAMS:2001:AD6753 (2001).

[4] Hartog ID Zomers M Van Thiel GJMW Leget C Sachs APE Uiterwaal CS Prevalence and Characteristics of Older Adults With a Persistent Death Wish Without Severe Illness: A Large Cross-Sectional Survey. BMC Geriatr (2020) 20(1):342. 10.1186/s12877-020-01735-0 32943009 PMC7495831

[5] Schnabel P Meyboom-de Jong B Schudel WJ Cleiren CPM Mevis PAM Verkerk MJ Voltooid leven. Over hulp bij zelfdoding aan mensen die hun leven voltooid achten. [Tired of Living. About Assisted Suicide for people Who Consider Their Lives complete] (2016). Available from: https://www.rijksoverheid.nl/documenten/rapporten/2016/02/04/rapport-adviescommissie-voltooid-leven2016 (Accessed April 5, 2023).

[6] Rurup ML Deeg DJH Poppelaars J Kerkhof AJFM Onwuteaka-Philipsen BD. Wishes to Die in Older People: A Quantitative Study of Prevalence and Associated Factors. Crisis-the J Crisis Intervention Suicide Prev (2011) 32(4):194–203. 10.1027/0227-5910/a000079 21940260

[7] Kox RMK Pasman HRW Heymans MW Benneker WHGM Onwuteaka-Philipsen BD. Current Wishes to Die; Characteristics of Middle-Aged and Older Dutch Adults Who Are Ready to Give up on Life: A Cross-Sectional Study. BMC Med Ethics (2021) 22(1):64. 10.1186/s12910-021-00632-4 34020628 PMC8140496

[8] Rurup ML Muller MT Onwuteaka-Philipsen BD Van Der Heide A Van Der Wal G Van Der Maas PJ. Requests for Euthanasia or Physician-Assisted Suicide From Older Persons Who Do Not Have a Severe Disease: An Interview Study. Psychol Med (2005) 35(5):665–71. 10.1017/s003329170400399x 15918343

[9] van der Heide A Onwuteaka-Philipsen BD van Thiel G van de Vathorst S Weyers H. Kennissynthese Ouderen en het zelfgekozen levenseinde (Knowledge synthesis: Older adults and the Self-Chosen End of Life). ZonMW (2014).

[10] Regionale Toetsingscommissie Euthanasie [Dutch Regional Euthanasia Review Committees]. EuthanasieCode 2018: De toetsingspraktijk toegelicht aangepast naar aanleiding van de arresten van de hoge raad van 21 april 2020. [The Dutch Euthanasia Code 2018: The assessment Practice Explained, Adjusted in Response to the Supreme Court Judgments of April 21, 2020] (2020). p. 1–70. Available from: https://www.euthanasiecommissie.nl/euthanasiecode-20182020 (Accessed April 7, 2023).

[11] Engelhart S Stall NM Quinn KL. Considerations for Assessing Frail Older Adults Requesting Medical Assistance in Dying. Can Med Assoc J (2022) 194(2):E51–E53. 10.1503/cmaj.210729 35039389 PMC8900794

[12] Evenblij K Pasman HRW Van Der Heide A Hoekstra T Onwuteaka-Philipsen BD. Factors Associated With Requesting and Receiving Euthanasia: A Nationwide Mortality Follow-Back Study With a Focus on Patients With Psychiatric Disorders, Dementia, or an Accumulation of Health Problems Related to Old Age. BMC Med (2019) 17(1):39. 10.1186/s12916-019-1276-y 30777057 PMC6379969

[13] Van Den BV Van Thiel GJMW Zomers M Hartog ID Leget C Sachs APE Euthanasia and Physician-Assisted Suicide in Patients With Multiple Geriatric Syndromes. JAMA Intern Med (2021) 181(2):245–50. 10.1001/jamainternmed.2020.6895 33284324 PMC7851730

[14] Van Wijngaarden E Van Thiel G Hartog I van den Berg V Zomers M Sachs A Het PERSPECTIEF-Onderzoek Perspectieven op de doodswens van ouderen die niet ernstig ziek zijn: De mensen en de cijfers [The PERSPECTIVE Study. Perspectives on the Death Wishes of Older People Who Are Not Seriously Ill: The People and the Numbers]. ZonMw: Den Haag (2020).

[15] Expertisecentrum Euthanasie [Center of Expertise Euthanasia]. Standpunten - Expertisecentrum Euthanasie [Points of View - Center of Expertise Euthanasia] (2022). Available from: https://expertisecentrumeuthanasie.nl/standpunten/2022 (Accessed April 7, 2023).

[16] Regionale Toetsingscommissie Euthanasie Dutch Regional Euthanasia Review Committees. Jaarverslag 2015 [Annual Report 2015] (2015). Available from: https://www.euthanasiecommissie.nl/de-toetsingscommissies/jaarverslagen2016 (Accessed June 11, 2023).

[17] Regionale Toetsingscommissie Euthanasie (Dutch Regional Euthanasia Review Committees). Jaarverslag 2022 [Annual Report 2022] (2022). Available from: https://www.euthanasiecommissie.nl/de-toetsingscommissies/jaarverslagen2023 (Accessed June 11, 2023).

[18] van der Heide A Legemaate J Onwuteaka-Philipsen B Bosma F Van Delden H Mevis P Vierde evaluatie Wet toetsing levensbeëindiging op verzoek en hulp bij zelfdoding (Fourth evaluation of the Termination of Life on Request and Assisted Suicide Act). Den Haag: ZonMw (2023). 16223080

[19] IQVIA. Medische Adressen IQVIA [Medical Addresses IQVIA] (2019). Available from: https://medische-adressen.nl/ (Accessed October 20, 2023).

[20] Van Wijngaarden E Leget C Goossensen A. Ready to Give up on Life: The Lived Experience of Elderly People Who Feel Life Is Completed and No Longer Worth Living. Soc Sci Med (2015) 138:257–64. 10.1016/j.socscimed.2015.05.015 25982088

[21] Humes LE Pichora-Fuller MK Hickson L. “Functional Consequences of Impaired Hearing in Older Adults and Implications for Intervention,” in Aging and Hearing: Causes and Consequences. Editor Helfer KS Bartlett EL Popper AN Fay RR (Cham: Springer International Publishing) (2020). 257–91.

[22] Davidson JG Guthrie DM. Older Adults With a Combination of Vision and Hearing Impairment Experience Higher Rates of Cognitive Impairment, Functional Dependence, and Worse Outcomes Across a Set of Quality Indicators. J Aging Health (2019) 31(1):85–108. 10.1177/0898264317723407 28805100

[23] Clynes MA Jameson KA Edwards MH Cooper C Dennison EM. Impact of Osteoarthritis on Activities of Daily Living: Does Joint Site Matter? Aging Clin Exp Res (2019) 31:1049–56. 10.1007/s40520-019-01163-0 30903599 PMC6661019

[24] Ten Cate K Van Tol D Van De Vathorst S. Considerations on Requests for Euthanasia or Assisted Suicide; a Qualitative Study With Dutch General Practitioners. Fam Pract (2017) 34(6):723–9. 10.1093/fampra/cmx041 28486577

[25] Pasman HRW Rurup ML Willems DL Onwuteaka-Philipsen BD. Concept of Unbearable Suffering in Context of Ungranted Requests for Euthanasia: Qualitative Interviews With Patients and Physicians. Bmj (2009) 339:b4362. 10.1136/bmj.b4362 19917578 PMC2777997

[26] Mroz S Deliens L Cohen J Chambaere K. Developments Under Assisted Dying Legislation: The Experience in Belgium and Other Countries. Deutsches Ärzteblatt Int (2022) 119(48):829. 10.3238/arztebl.m2022.0378

[27] Healthdirect Australia. Voluntary Assisted Dying 2023 (2023). Available from: https://www.healthdirect.gov.au/voluntary-assisted-dying (Accessed April 12, 2023).

