# Supplementary material for: Euthanasia and Physician-Assisted Suicide in People With an Accumulation of Health Problems Related to Old Age: A Cross-Sectional Questionnaire Study Among Physicians in the Netherlands
Source: Int J Public Health. 2024 Apr 18;69:1606962. doi: 10.3389/ijph.2024.1606962 (PMC11064696; doi:10.3389/ijph.2024.1606962)
Supplement: Supplementary file 1 [file DataSheet1.docx]

**Additional files**

**Additional file 1. (The Netherlands. 2022)**

If physicians only had experience with an EAS request of a person with dementia or both dementia and another condition (not an accumulation of health problems related to old age), they were directed to the version on dementia. If physicians only had experience with an EAS request of a person with an accumulation of health problems related to old age or both an accumulation of health problems related to old age and another condition (not dementia), they were directed to the version on an accumulation of health problems related to old age. If physicians had both experience with an EAS request of a person with dementia and of a person with an accumulation of health problems related to old age, they were randomly assigned to the version on dementia or the version on an accumulation of health problems related to old age. If physicians only had experience with an EAS request of a person with another condition and not with dementia and an accumulation of health problems related to old age, they were directed to the version on another condition.

**
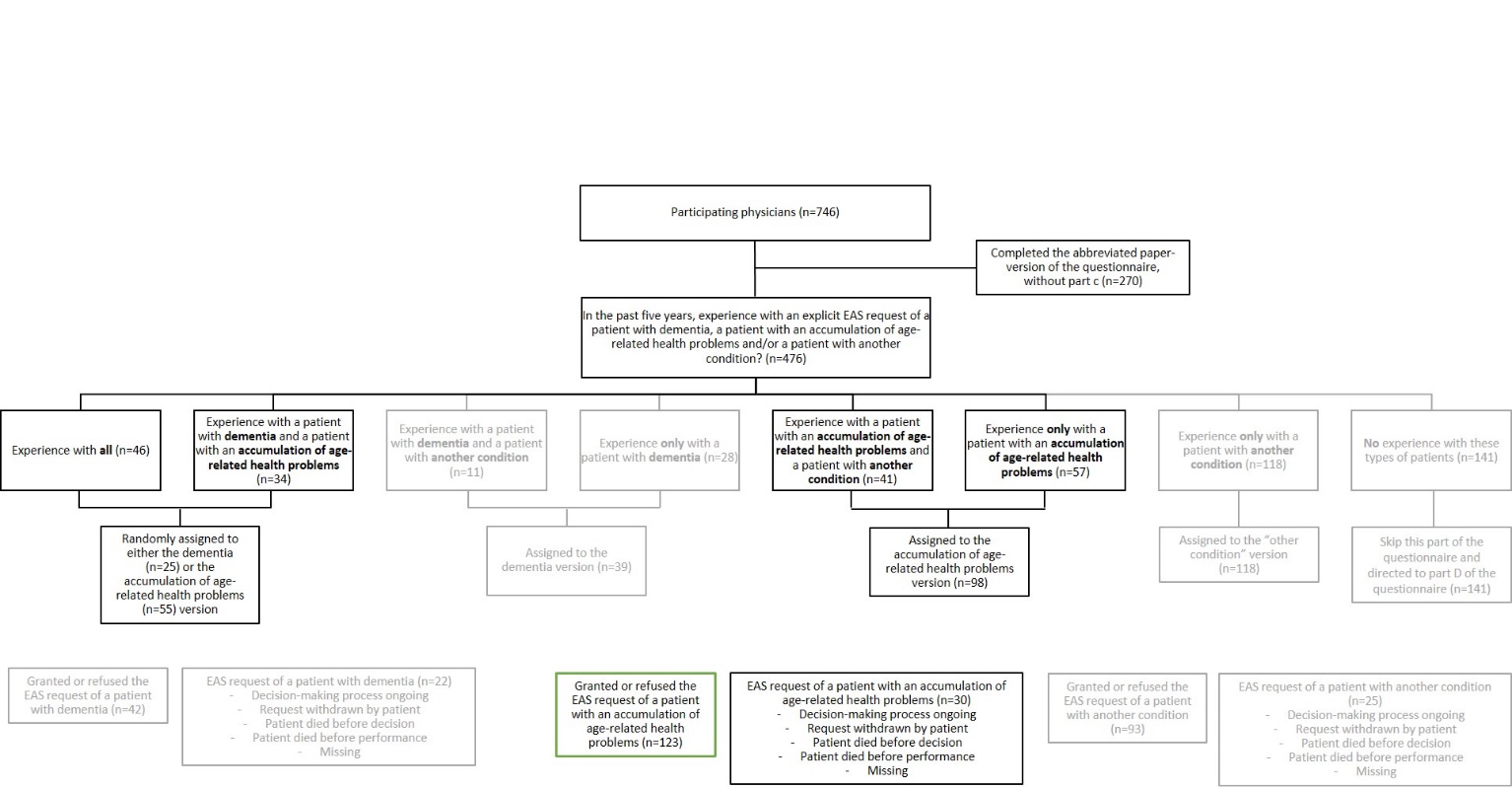
**

**Additional file 2**. Implemented variables (The Netherlands. 2022)

1. Characteristics of the physician

| **Variable** | **Question/items** | **Answer categories** | **Recode** |
| --- | --- | --- | --- |
| Age | ‘What is your age?’ | Continuous (in years) | Categorized into:   1. < 41 years 2. 41-50 years 3. 51-60 years 4. > 60 years |
| Gender | ‘What is your gender?’ | 1. Male 2. Female 3. Other | n.a. |
| Religion | ‘Do you have a religion?’ | 1. Yes 2. No | n.a. |
| Medical specialty | ‘What is your medical specialty?’ | 1. General practitioner 2. Elderly care physician 3. Internist 4. Cardiologist 5. Surgeon 6. Pulmonologist 7. Neurologist 8. Intensivist 9. Other, namely… | Categorized into:   1. General practitioner 2. Elderly care physician 3. Clinical specialist |
| Years of work experience in medical specialty | ‘How many years have you been working as a physician?’ | Continuous | Categorized into:   1. < 11 years 2. 11-20 years 3. 21-30 years 4. > 30 years |
| Palliative care consultant | ‘Do you work as a palliative care consultant?’ | 1. No 2. Yes | n.a. |
| SCEN physician | ‘Do you work as a SCEN physician?’ | 1. No 2. Yes | n.a. |
| Certified palliative care training | ‘Did you receive (certified) palliative care training (in addition to your regular training)?’ | 1. No 2. Yes | n.a. |
| Number of deceased patients in the last year | ‘How many patients who were under your care died in the past year?’ | Continuous | Categorized into:   1. < 10 patients 2. 10-14 patients 3. 15- 25 patients 4. > 25 patients |

1. Experiences with EAS requests and their performance

| **Variable** | **Question/items** | **Answer categories** | **Recode** |
| --- | --- | --- | --- |
| Ever consulted the Dutch Euthanasia Code | ‘How often do you consult the Dutch Euthanasia Code?’ | 1. With every EAS request 2. For some EAS requests 3. Never | Categorized into:   1. No 2. Yes (answer options 1+2) |

1. Last request within the past 5 years for EAS from a patient with an accumulation of health problems related to old age

| **Variable** | **Question/items** | **Answer categories** | **Recode** |
| --- | --- | --- | --- |
| Age | ‘What was the age of the patient at the time EAS was performed/declined?’ | Continuous (in years) | Categorized into:   1. < 80 years 2. 80-89 years 3. > 90 years |
| Gender | ‘What was the gender of the patient?’ | 1. Male 2. Female | n.a. |
| Health problems related to old age | ‘Which health problems related to old age did the patient have?’ | 1. Vision impairment 2. Hearing impairment 3. Osteoporosis 4. Osteoarthritis 5. Balance problems 6. Cognitive decline 7. Other | n.a. |
| Main reason/reasons for EAS request | ‘What were the patient's main reasons for making the explicit EAS request?’ | 1. General weakness/fatigue 2. Dyspnea 3. Pain 4. Other physical complaints 5. Depressed feelings 6. Fair 7. Cognitive decline 8. Physical decline 9. Disability/immobility 10. Death of a loved one 11. Loneliness 12. Dependency 13. (Fear of) losing control of one’s own life 14. Not wanting to be a burden to family/environment 15. No longer living independent 16. Suffering with no prospect of improvement 17. No purpose in life 18. Loss of dignity 19. Tired of living 20. Other | n.a. |
| Partner | ‘Did the patient have a partner at the time EAS was performed/declined?’ | 1. Yes, living together 2. Yes, not living together 3. No, widow/widower 4. No , other | Categorized into:   1. No 2. Yes (answer options 1+2) 3. Widow/widower |
| Place of residence last 3 months | ‘Where did the patient reside the longest in the last 3 months of his/her life?’ | 1. At home or with a loved one 2. In a hospital 3. Hospice 4. Nursing home or care home | Categorized into:   1. At home or with loved ones 2. Institutionalized (answer options 2-4) |
| Dependency | ‘To what extent was the patient dependent on others at the time EAS was performed?’ | 1. Independent 2. Limited care dependent 3. Care-dependent | n.a. |
| Life-expectancy | ‘What was the patient's life expectancy at the time EAS was performed/declined?’ | 1. Less than 1 week 2. 1-4 weeks 3. 1-5 months 4. 6-12 months 5. More than 12 months | Categorized into:   1. Up to 12 months 2. More than 12 months |
| Duration of treatment relationship | ‘How long was the patient under your care at the time EAS was performed/declined?’ | 1. Less than 1 month 2. 1-12 months 3. More than 12 months | Categorized into:   1. Up to 12 months 2. More than 12 months |
| Period between first EAS conversation and explicit request | ‘How long before the explicit EAS request did the first conversation about EAS with the patient occur?’ | Continuous (in days, weeks, months or years) | Categorized into:   1. < 1 month 2. 1-6 months 3. 7 months-1 year 4. > 1 year |
| Duration of the decision-making process | ‘How long did the decision-making process on this EAS request take from the explicit request until you performed the EAS?’ | Continuous (in days, weeks or months) | Categorized into:   1. Up to 12 months 2. More than 12 months |
| EAS performed/request for EAS declined | ‘What did you do with this last explicit request?’ | 1. Granted 2. Declined 3. Decision-making still ongoing 4. Patient withdrew the request 5. The patient died before I made the decision on the request 6. The patient died after I granted the request but before I could perform the EAS | In this study, only the cases where option 1 or 2 were included. |
| Performed euthanasia or physician-assisted suicide | ‘Did you perform euthanasia or assisted suicide?’ | 1. Performed euthanasia 2. Assisted in suicide | n.a. |
| Reasons for declined EAS | ‘What was the main reason for denying the request?’ | 1. I never perform EAS 2. Objections from family 3. Possible failure to meet due care criteria 4. Personal objections specific to this case 5. Other | n.a. |
| Treatment after declined EAS | ‘What decisions regarding treatment and care were made after refusing the EAS request?’ | 1. No change in treatment 2. Curative treatment stopped 3. Symptom management 4. Discussed treatment limitation 5. Psychological counselling 6. Expansion of social activities 7. Expansion of care 8. Palliative sedation 9. Transfer to hospice 10. Transfer to other care facility 11. Discharge home 12. Home care started/expanded 13. Other | n.a. |
